# MULTIGENERATIONAL DYNAMICS AND FUTURE CARE PLANNING: A QUALITATIVE STUDY OF THREE-GENERATION FAMILIES IN HONG KONG

**DOI:** 10.1093/geroni/igae098.3869

**Published:** 2024-12-31

**Authors:** Xue Bai, Rui Kang, Ke Ma, Kun Tian, Jing Hu

**Affiliations:** The Hong Kong Polytechnic University, Hong Kong, Hong Kong; The Hong Kong Polytechnic University, Hong Kong, Hong Kong; The Hong Kong Polytechnic University, Hong Kong, Hong Kong; The Hong Kong Polytechnic University, Hong Kong, Hong Kong; The Hong Kong Polytechnic University, Hong Kong, Hong Kong

## Abstract

Numerous ageing adults need to provide care for older and/or younger generations while also facing life challenges associated with the transition to old age. However, no systematic investigation has examined aging sandwich carers’ future care planning, nor the impacts of intergenerational care provision on their care planning. A purposive sample of 120 individuals from 40 multigenerational families in Hong Kong was recruited through various channels. The families all filled the following inclusion criteria: (a) had at least one adult aged 50–69 years with both surviving parents and children, (b) all three generations were willing to participate, and (c) the participants spoke Chinese and lived in Hong Kong. For each eligible family, an older parent from the oldest generation, an adult from the sandwiched, and a child from the youngest generation were interviewed separately. Both inductive and deductive coding approaches were used for data analysis. The findings reveal that lifelong multigenerational care transfer and intergenerational relationships shaped the future care planning and preparation processes and experiences. The upward care provision and substantial investment in child-rearing often lead to delayed and indirect reciprocity expectations from the sandwich generation. Moreover, the experience of caring for aging parents prompts sandwich generation to make financial, health, and social life-related care plans. However, traditional family communication patterns often obstruct open discussions about care plans between sandwich generation and their children. Anticipated future circumstances of both aging parents and children further shape the intended care arrangements of the sandwich generation.

